# Selected Quality Attributes of Freshwater Mussel Powder as a Promising Ingredient for Pet Food

**DOI:** 10.3390/ani12010090

**Published:** 2021-12-31

**Authors:** Piotr Konieczny, Wojciech Andrzejewski, Tianyu Yang, Maria Urbańska, Jerzy Stangierski, Łukasz Tomczyk, Beata Mikołajczak

**Affiliations:** 1 Department of Food Quality and Safety Management, Faculty of Food Science and Nutrition, Poznan University of Life Sciences, Wojska Polskiego 31, 60-624 Poznan, Poland; tianyu.yang@up.poznan.pl (T.Y.); jerzy.stangierski@up.poznan.pl (J.S.); lukasz.tomczyk@up.poznan.pl (Ł.T.); 2 Department of Zoology, Division of Inland Fisheries and Aquaculture of Zoology, Faculty of Veterinary Medicine and Animal Science, Poznan University of Life Sciences, Wojska Polskiego 71C, 60-625 Poznan, Poland; urbanska@up.poznan.pl; 3 Department of Meat Technology, Poznan University of Life Sciences, Wojska Polskiego 31, 60-624 Poznan, Poland; beata.mikolajczak@up.poznan.pl

**Keywords:** freeze drying, mussel, novel materials, *Sinanodonta woodiana*, soft tissues, pets

## Abstract

**Simple Summary:**

Bivalves such as clams, mussels, and oysters provide a good source of protein, glycogen, and minerals. The high-value compounds and bioactive properties of these organisms are well known and confirmed by numerous scientific studies. During the last two decades, the freshwater mussel *Sinanodonta woodiana* as one of the most invasive species, has penetrated into Europe along with fish shipments from Asia. Numerous studies indicate that both the soft tissues and mussel shells of this low-grade species may constitute promising biomaterial with potential processing ability. Due to growing interest in new raw materials having been observed recently in the pet food market, this study aimed to obtain and evaluate selected quality attributes of freshwater pond mussel powder. After initial ecotoxicity testing in vitro, powdered mussel tissue was evaluated with respect to its basic physicochemical and functional properties, such as solubility, emulsifying capacity, and gel-forming ability. The study also provides novel information about electrophoretic protein profile and amino acid composition, including taurine content. Based on the results obtained in this study, it can be concluded that tissues of mussels available locally from lakes and ponds can be proposed as an interesting source of new ingredients for developing formulated pet food products.

**Abstract:**

The aim of this study was to describe the quality attributes of a freeze-dried preparation obtained from freshwater mussel *Sinanodonta woodiana* (SW) soft tissue in respect to its potential as a novel pet food ingredient. After ecotoxicological testing of the raw material with MARA (Microbial Assay for Risk Assessment), the basic physico-chemical properties of the powder, such as approximate composition, bulk density, color parameters, water activity, electrophoretic analysis (SDS-PAGE), solubility, gelling and emulsifying capacity, were analyzed. The powder with a water activity of 0.43 offers a toxically safe preparation that contains over 34% protein/100 g of dry matter (DM). The SDS-PAGE profile showed twelve protein bands with a molecular weight (MW) ranging from >250 to 10 kDa. Taurine content has been estimated at an essential amount above 150 mg/100 g of DM. The powder possessed desirable emulsifying properties with 230 mL per 1 g and demonstrated the ability to form a firmer gel with a strength of 152.9 g at a temperature above 80 °C with at least 10% protein content. The L*, a*, and b* values characterizing powder color were found to be 69.49, 16.33, and 3.86, respectively. The SW mussel powder seems to be a promising ingredient that can be added with other binding or gelling agents in order to improve both the taste and acceptance of the final pet food products.

## 1. Introduction

In the last several years, the global pet food market has shown a rising trend along with the economy and the consumption recovery. This market was worth US $98.3 billion in 2018 and grew at a CAGR (Compound Annual Growth Rate) of 5.3% in the period 2011–2018. The market is projected to reach a value of US $128.4 billion by 2024 by growing at a CAGR of 4.5% during 2019–2024 [1]. Global pet food production expanded by 8% in 2020 to 29.33 million metric tons, which was driven primarily by growth in Europe [2]. For comparison, in China, the pet food market was up to $1.3 billion, and many citizens kept pets, focusing currently more on commercially available products for pets [3].

The pet food industry is regularly innovating and developing new formulas and products. Many examples are known of by-products of human food sources being used as raw materials for pet food purposes [4,5,6]. On the basis of ingredients, the reports mentioned above find that animal-derived pet food is the leading segment, followed by plant-derived pet food. More and more researchers are trying to find different functional, high energy, and cost-effective ingredients, like beans, peas, and other pulses, miscanthus, etc. Recognizing the nutritional benefits of functional foods currently available is of key importance to providing dogs and cats with the relevant diet to keep them healthy [4,6,7].

Recently, researchers have paid increasing attention to seafood, and not only fish species but also mollusks such as mussels (Bivalvia), blue mussels (Mytilidae), or green mussels (*Perna viridis*). Two decades ago, green-lipped mussels had already been discovered to be suitable for treating arthritic pain [8], while their freeze-dried preparation reduced osteoarthritis symptoms in dogs [9]. It is worth adding, a novel green-lipped mussel extract has also been developed for use in managing pain in moderate to severe osteoarthritis of the hip and knee affecting humans [10]. S. *woodiana* (SW) is a large species of mussel that is recognized as a species native to East and South-East Asia. This species is widely distributed across China and is commonly used for food and for cultivating pearls. As an invasive species, alien individuals that were transported with fish shipments penetrated from Asia to Europe, including Poland [11,12]. In any case, freshwater mussels have been identified as a safe and interesting raw biomaterial for both food and non-food applications [13,14,15].

The review by Chakraborty et al. [16] provided a comprehensive summary of the nutritional qualities, functional attributes, and high-value bioactive compounds of the mollusks of marine and estuarine ecosystems. The report by Zhang et al. [17] confirmed the high quality of freshwater mussel tissues collected in China as a pearl production leftover. Studying the operating parameters of spray-drying in a series of preprocessing experiments and selecting quality attributes of the obtained preparations, the authors finally concluded that the mussel powder offers an acceptable edible resource with high nutritional quality for humans. Based on the results obtained, the mussel powder could be eaten directly or mixed with other functional products.

Therefore, the information presented above inspired our team to prepare the invasive *S. woodiana* freshwater mussel powder as a new proteinaceous substance by the freeze-drying method, which can be potentially applied to improve the functionality of pet food. This preliminary study introduces an analysis of selected quality attributes of freeze-dried mussel powder. The value of the raw material is not yet been commercially evaluated, but, anyway, it seems that it can be considered as a potential ingredient for typical pet food for both cats and dogs.

## 2. Materials and Methods

### 2.1. Preparation of Freshwater Mussel Powder

Freshwater mussels were collected from Lake Goslawskie. Located in Central Poland, this is one of several shallow lakes known collectively as the heated ‘KoninLakes’ because the water temperature is raised as a result of the outflow received from two power stations.

The hand gathered mussel bodies were dissected from their shells and frozen at a temperature of approximately −22 °C. Before the start of the drying process, the samples were defrosted at a temperature of 4–6 °C for approximately 12 h. The slightly frozen mass was minced for 2 min. in a lab blender (Bosch, Berlin, Germany) before being frozen at −50 °C. Freeze-drying was carried out in a freeze-dryer (Labconco, Kansas City, MO, USA) at a pressure of 13.3 Pa for 48 h at a shelf temperature of 22 °C (±1 °C) in the final product.

### 2.2. Procedure of Microbial Assay for Risk Assessment (MARA)

A commercially available array of eleven genetically diverse microorganisms, freeze-dried in a microtiter plate, was used for the initial ecotoxicity evaluation of the mussel powder obtained in this study. MARA (Microbial Assay for Risk Assessment) was a 24-h multi-species assay used to measure the ecotoxicity of chemicals and environmental samples. The taxonomically diverse microbial species used in the test were lyophilized on a microplate. The microbes consisted of 10 prokaryotic species and one eukaryote (yeasts). Table 1 shows the details of the microorganisms used [18]. In the assay, the microorganisms were exposed to a dilution series of the samples, and the growth of the microorganisms was established with the reduction of tetrazolium red (TZR) [19].

**Figure 1:**
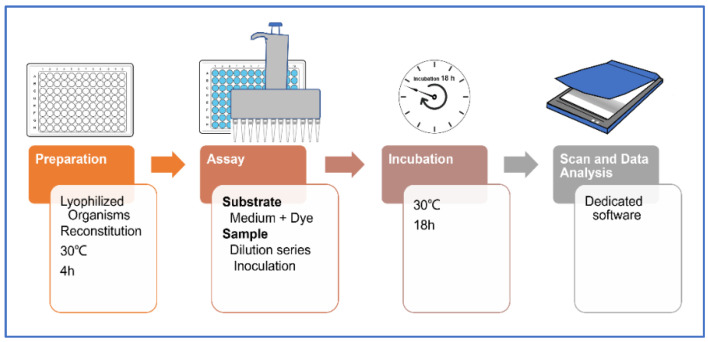
MARA framework.

A scanned image of the microplate was obtained using a flatbed scanner, and this was then subjected to analysis using purpose-built software. The system framework is presented in Figure 1 adopted from the report of Wadhia, 2013 [20].

The powder sample (4.0 g) was shaken in distilled water (40 mL) overnight at ambient temperature using an Incu-Shaker (Benchmark Scientific), and centrifuged for 15 min at 4000 rpm. A total of 20 mL of supernatant was collected using a syringe with a 0.45 µm filter. Then 0.4 g of phytone peptone was dissolved in the sample, and 200 µL of tetrazolium red (TZR). In line with the general procedure [18,19,21], the array was exposed to a concentration gradient of the test sample in the presence of a growth medium. The growth of the microorganisms in the bioassay at each sample dilution was measured by recording the reduction in TZR. After incubation, the test plates were scanned by means of a flatbed scanner. The image obtained was subsequently analyzed using purpose-built software to provide a unique fingerprint of the sample, which was expressed in terms of the sensitivity of the ten constituent prokaryotic and one eukaryotic (yeast) species. Sample toxicity assessment using MARA is conducted with regard to the microbial toxic concentration for each microbial species and the overall assay mean.

To provide a comprehensive and optimal assessment utilizing the significant feature of the MARA as a multi-species test, a determination referred to as the Microbial Toxic Concentration (MTC) is computed. The MTC value is determined thus:

MTC = c_min_ × d^(Ptot/Po)−1^ (1)

where: c_min_ = the lowest concentration in the gradient, P_o_ = pellet size in the control, d = dilution factor and P_tot_ = six pellet sizes down the concentration gradient. The MTC values for the MARA are generated for each species. The MTC value is the endpoint calculated by the MARA software, on the basis of raw data about the inhibition of each microorganism’s growth. Moreover, a single value for the assay as a whole is also determined. The MARA toxic fingerprint (FIP), as uniquely characteristic of the examined mussel powder sample and potentially indicative of the mode of toxic action, has been applied in this study.

### 2.3. Muscle Protein Electrophoresis (SDS-PAGE)

The electrophoretic protein separation (SDS-PAGE) of pond mussel powder was conducted in 15% polyacrylamide two-phase gels (30% (*w*/*v*) acrylamide, 75% (*v*/*v*) glycerol, 3 M Tris [pH 8.8], 10% (*w*/*v*) SDS, 1% (*w*/*v*) ammonium persulfate, and 16 µL of TEMED and 10% (*w*/*v*) acrylamide, 5% (*v*/*v*) glycerol, 0.125 M Tris (pH 6.8), distilled water, 10% (*w*/*v*) SDS, 1% (*w*/*v*) ammonium persulfate and 25 µL of TEMED). The proteins were separated in tris-glycine buffer pH. The samples (12 µg of protein) were applied to the gel prepared in triplicate. The evaluation of the gels was performed by the ImageMaster^®^ VDS system and developed using ImageMaster^®^ 1DElite v.4.0 (Pharmacia Biotech, Vienna, Austria). All peaks marked on the gel were 100% in total. The molecular weight of selected mussel proteins was determined based on the standard in the range of 10–250 kDa (PageRuler Plus Protein Ladder, Thermo Scientific, Waltham, MA, USA) [22].

### 2.4. Physicochemical Properties and Amino Acid Composition

The freshwater mussel powder was analyzed for its proximate composition as follows: the moisture content was established by drying in an oven at 100 °C to a constant weight, crude protein was determined indirectly from the analysis of total nitrogen (crude protein = N × 6.25) by the Kjeldahl method, and lipid content was established after Soxhlet extraction of dried samples with 1.25% H2SO4 and 1.25% NaOH. Ash was determined from weighed samples in a porcelain crucible placed in a muffle furnace at 550 °C for 6 h. The carbohydrate content of a powder was determined by calculating the percent remaining after all the other components had been measured [23].

Among other mussel powder properties, the bulk density was determined by pouring approximately 5 g of the powder into a 10 mL graduated cylinder. The volume occupied by the sample was recorded and bulk density was calculated [17].

The color measurement on sample trials was carried out with a MINOLTA KONICA CM-5 spectrophotometer (Japan) in CIE L*, a*, b* scale. The equipment was calibrated and standardized with a white color standard calibration plate as proposed by the manufacturer. Parameters of the device in the transmission mode were set as follows: a standard observer of 10° and an illuminant D65. The color was measured at room temperature using mussel powder placed in plastic Petri dishes and the color measurement was repeated at least three times [17].

The water activity measurement was determined by a test instrument (Rotronic 8303 Bassersdorf, Swiss). The powder was tested in three replications and expressed as water activity units in the range between vales 0 and 1 [17].

Due to the method recommended by the Association of Official Analytical Chemists (AOAC) [23], the amino acids in the mussel powder protein were separated after being hydrolyzed in 6n HCl at a temperature of 105 °C for 24 h on AAA-400 (INGOS, Prague, Czech Republic). Sulfur amino acids, methionine, and cystine were determined after they had been oxidized and fixed with formic acid. Finally, the amino acid composition was expressed in g amino acids per 100 g of protein. No analysis was carried in reference to tryptophan.

Furthermore, taurine content was determined in the local HAMILTON Ltd. laboratory using high performance liquid chromatography (HPLC) with an external standard fluorescence detector, as published and adopted from National Food Safety Standard China GB 5413.26—2010 [24].

### 2.5. Solubility

Water solubility was determined by measuring the insoluble particles present in a solution of the powder in water following the method described by Zahng et al. [17], with some slight modifications. In brief, 0.5 g of the powder was dissolved in 5 mL of distilled water. The tubes holding the solution were centrifuged in a laboratory centrifuge at 3000 rpm for 15 min. After centrifugation, the liquid was discarded, and the pellet remaining at the bottom of the tubes was measured after having been dried at 105 °C overnight. Thus, the solubility of the powder was calculated by determining the percentage of insoluble material with respect to the initial quantity dissolved.

### 2.6. Least Gelation Concentration (LGC) and Gel Strength Analysis

The Least Gelation Concentration (LGC) of the mussel powder was determined according to the method described by Uzzaman et al. [25] with slight modifications. In 10mL of distilled water, powder suspensions of 7%, 8%, 9%, and 10% (*w*/*v*) were prepared. The tubes containing the suspensions were then heated for 30 min in a gentle boiling water bath, after which the tubes were cooled rapidly in water at 4 °C for 60 min. Each tube was then inverted, one after the other. The LGC was taken as the concentration when the sample from the inverted test tube did not fall or slip.

The TA-XT2i analyzer (Stable Microsystem, Surrey, UK) was used to analyze the single compression test for the selected gels. In the test, a cylindrical plexiglass sensor with a diameter of 1.27 mm (P/0.5R, Stable Microsystem, UK) was pushed into the sample at a velocity of 0.5 mm × s^−1^. The samples were penetrated to a depth of 30 mm. The measurements were taken at a rate of 200 measurement points per second (PPS 200) with a trigger force of 0.05 N. Then, the maximum force obtained during the first compression cycle was expressed in N × cm^−2^ of the probe area [26].

### 2.7. Fat Emulsifying Capacity

Fat emulsifying capacity is the property of keeping a homogeneous mixture of oil and the sample solution. In this method, 0.1 g of SW powder and 0.1 g of plasma as a reference sample were dissolved separately in the 0.9% NaCl solution. The soya oil was added dropwise into a sample solution from 55 mL burettes and stirred vigorously (using an ULTRA-TURRAX T25 helix stick at 5000 rpm). The emulsifying capacity value was determined at the inversion point at which an oil-in-water emulsion turns into a water-in-oil emulsion, as indicated by a sudden drop in conductivity. The emulsifying capacity is expressed as ml of oil per 1 g of product [26,27].

### 2.8. Statistical Analyses

In general, analysis of physicochemical properties was conducted at least in triplicate, calculating mean values and the standard deviation (SD). The resulting data are presented as mean values ± SD.

## 3. Results and Discussion

A total of 30 living individuals of the *S. woodiana* mussel collected as a random sample from the lake were analyzed in this study. Before proceeding with the experiment, the mussels’ biometric data were determined: shell length ranged between 109 and 153 mm, shell height ranged between 42 and 71 mm, and shell width ranged between 77 and 105 mm. The individuals were characterized by total wet weight of between 195.3 and 549.0 g, respectively. Selected quality and safety attributes of the research material were analyzed in this study after its successful freeze-drying as described above.

### 3.1. Evaluation of Mussel Powder Toxicity by MARA Assay

In order to ensure the safety of the raw material, the initial aim of this study was to identify its ecotoxicity, reflecting the degree to which the mussel soft tissue was contaminated with selected chemical contaminants like heavy metals, organochlorine chemicals, and other pollutants [18].

In general, in line with the methodological recommendations published by Wadhia et al. [19], the MARA assay measurements are performed on various environmental samples diluted with distilled water. Data from the analysis of a sample is obtained as:

- the minimum MTC value is equal to the concentration that is toxic to the most sensitive species,
- the maximum MTC value is equal to the concentration that is toxic to the least sensitive species,
- the mean MTC value is equal to the mean of the concentrations that are toxic to all species,
- the toxic fingerprint (FIP) as an array of MTC values for all tested strains.

In our study, for a better comparison of the FIP of examined powder, they are shown in a specially prepared chart. For this purpose, the MTC values have been converted into units of toxicity: TU = 100/MTC, and then the percentage TU was calculated for each strain by taking the TU value for the most sensitive strain as 100% [28].

**Figure 2:**
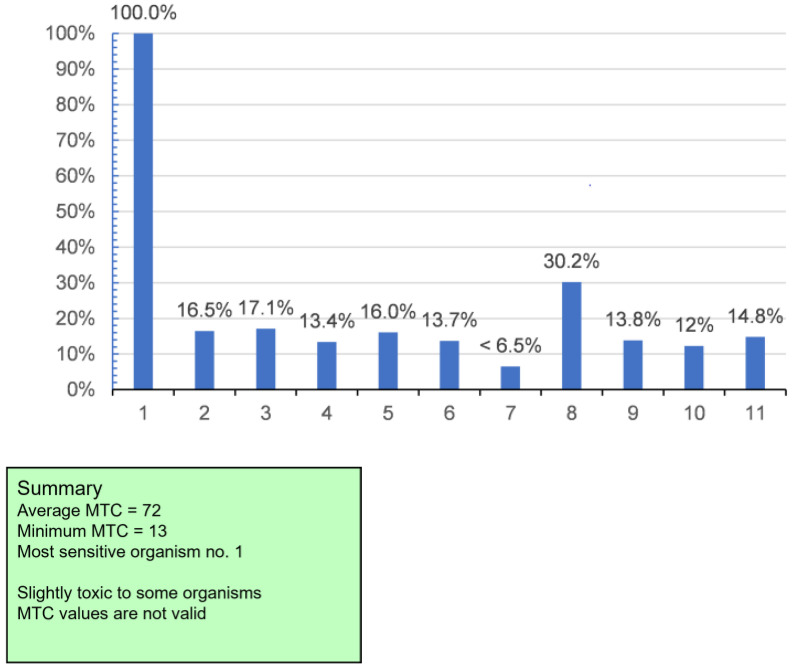
Finger print (FIP) of tested mussel powder, strain with y-axis 100% indicates most sensitive organism (description of 1 to 11—see Table 1).

The result of the MARA test is summarized graphically in Figure 2. Based on the MARA result, freeze-dried mussel powder was categorized by internal system software as non- or extremely low-toxic.

### 3.2. Proximate Composition and Selected Physicochemical Properties of Tested Powder

Table 2 demonstrates the basic chemical properties of *S. woodiana* (SW) powder. Compared with other animal raw protein-based materials [4,29], it was recognized as a good nutritional source. When compared to fresh soft tissue, which had a crude protein content of more than 65% in dry matter (DM), the freeze-dried powder had a protein content of more than 34% in 100 g DM. The results show that the SW powder was also a good source of carbohydrates (52.22%). Zhang et al. [17] reported a relatively high content of this compound, determined as glycogen, of 27.71% in spray-dried mussel powder. It should be stressed that in the work presented here, some parts of mussel bodies were not discarded during the preparation process. Therefore, it is likely that some foods of mussels, such as algae, rotifers, or plant origin materials, were inside the stomach, which affected the final result of this parameter. Additionally, according to Królak et al. [12], the living environment and water quality can also influence the discrepancies in nutrition content observed in this study.

Among the conditionally indispensable amino acids, taurine has attracted attention due to its suggested strong contribution to the health-promoting effects of seafood [30,31,32]. Taurine is an essential amino acid in cats. Taurine is an essential nutrient in cats’ diets and may be conditionally essential for dogs. Cardiac function, eye health, immune system function, and other body systems are affected by taurine availability. The freshwater *S. woodiana* soft tissue powdered by freeze-drying with the content of taurine at a level of 150 mg/100 g DM seems to be a relatively rich ingredient, and this observation corresponds well with previous studies [30,31]. Generally, animal muscle tissue, particularly marine muscle tissue, contains high taurine concentrations. Fresh seafood was found to contain the highest concentrations of taurine. Plant products contain either low or undetectable amounts of taurine. The impact of cooking or drying on the taurine content in processed seafood and their corresponding unprocessed raw materials should be taken into consideration during any diet formula development [32].

A healthy diet should guarantee an adequate intake of all the essential amino acids (EAAs) in order to ensure metabolic functions, normal growth, and maintenance of the animal. Three AAs: isoleucine, leucine, and valine, also called branched-chain amino acids, are of particular importance [4,7]. The meat tissue of marine and freshwater bivalves is often recommended as a high-quality protein source with essential protein levels. In general, the protein possesses a similar amino acid profile to fish meal [33,34,35,36]. Also, in the case of the powdered freshwater mussel soft tissue examined and compared in this study together with typical commercially made fish meal as reported by Vidakovic, 2015 [37] (Table 3), the amino acid composition of mussel powder has confirmed its general potential as an interesting compound in respect to diets for nutritional purposes [17].

**Figure 3:**
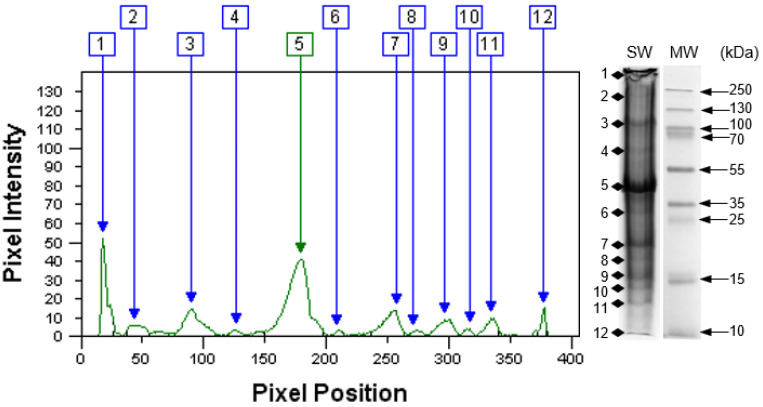
Electrophoretic profile of proteins *S. woodiana* (SW) mussel powder. 1–12 bands of proteins. MW—molecular weight of protein (kDa) standard.

Figure 3 presents the electrophoretic (SDS-PAGE) separations of proteins from mussel powder. Twelve easily visible bands of proteins for SW samples were identified. They correspond to proteins with a molecular weight (MW) in the >250 and 10 kDa range. The MW and percentage share of the marked protein bands are shown in Table 4. The results of protein SDS-PAGE profiles show that in total. The share of band 1 (>250 kDa), paramyosin (98–107 kDa), and actin (43–46 kDa) was over 63%. The highest share, approximately 39%, was characteristic of protein band 5 corresponding to actin of 43–46 kDa. In our own research, the proportion of myosin (band 2) was small (4.55%). Although the band with a weight above 250 kDa may contain: myosin-heavy chains (MHC), its degradation products and aggregates with other proteins. A previous study of raw muscles of *S. woodiana* showed a greater proportion of MHC [13]. In this study, the share of MHC, together with paramyosin and actin, accounted for over 50% of the total and had the greatest impact on the denaturation curve DSC. The scallop adductor muscle is attached by a small piece of smooth muscle, which is high in paramyosin. It has been found that the content of paramyosin in smooth muscle is significantly greater than that of myosin [38]. The resulting SW preparation offers a different protein profile compared to raw muscle [4], especially with respect to proteins with a molecular weight > 130 kDa. The above result also indicates the formation of lower molecular weight peptides (<10 kDa), which may be hypoallergenic and more digestible [6].

Among physicochemical attributes, the water activity of the powder being tested was found to be equal to 0.43 (Table 5), which is less than most dried food products, typically demonstrating a water activity value of approximately 0.65. Drying to reduce water content and water activity is a common way to increase the shelf life of food products [27]. The bulk density of the preparation examined was only 0.32 mg/L, much lower in comparison to the data presented by Zhang et al. [17] for spray-dried mussel preparations ranging from 0.55 g/mL to 0.62 g/mL. This parameter was affected by the drying method of preparation. This is an important property of any powdered protein preparation used in the food and feed industry [27].

It has also been shown that the color of the *S. woodiana* preparation powder was not white, but rather a light darker, moving towards light brown. The data show a wide range of powder quality variations with respect to all instrumentally determined color parameters such as L*, a*, and b*. It seems reasonable to point out that color optimization of *S. woodiana* powder could still be a subject for separate studies. In general, the dark color of ingredients used for pet food production should be avoided due to currently observed preferences among customers/owners of animals [5].

The analyses performed in this study also included a description of solubility, gelling, and emulsifying abilities, as suggested and reported by many authors studying the quality attributes of various animal and plant origin preparations [17,26,27].

Under selected analytical conditions, the relatively low solubility of the powder being tested (below 50%) was determined. Zhang et al. [17] reported a solubility range of spray-dried mussel powder between 46.55 and 51.27% for preparations obtained in their experiments by the use of various drying temperatures.

A minimum protein concentration is necessary for gelation. Low protein concentrations based on intramolecular protein interactions fail to form a gel structure. The minimal protein concentration required to form a gel is ascertained using the LCE (Least Concentration Endpoint) test procedure, which has been developed to provide useful comparisons between the gelling properties of different preparations and conditions. Factors of importance in the gelation process include protein hydrophobicity, pH, salt, calcium, and free sulfhydryl concentrations. Additionally, residual lipids are expected to inhibit the gelation of proteins [27].

From the results of this study, it appeared *that S. woodiana* powder prepared by the freeze-drying method demonstrated some gel-forming ability, which was affected by the heating temperature and concentration of the protein preparation. According to the data in Table 6, it was found that 80 °C was the recommended gelation temperature. While for this temperature, the 10% concentration of protein in the solution was selected as the LGC value. For comparison, spray-dried tilapia powder was used in the study by Uzzaman et al. [25]. The lowest gelation concentration was found to be 8.67%, and it was lower than that obtained for plant origin preparations like Great Northern bean (10%). lupin seed flour (14%). cowpea flour (16%).and pigeon pea flour (12%). However, there is no evidence that the authors cited above used the same analytical procedure during their studies.

**Figure 4:**
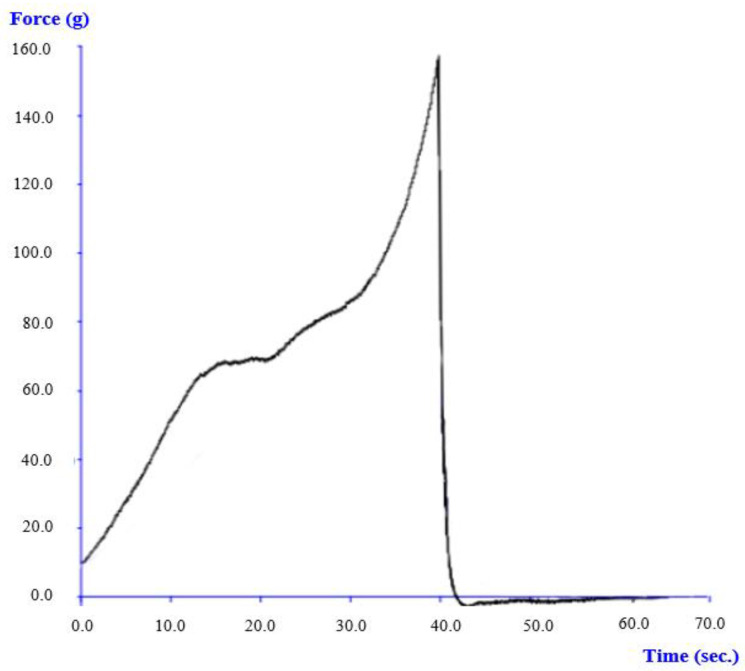
Force penetration curve for SW gel at 10% protein concentration (*n* = 3).

Gels prepared from *S. woodiana* protein solutions at 10% *w*/*v* had an appearance similar to that of other proteins of animal or plant origin [25,26]. A penetration test was used to characterize the texture of the 10% gel. As the force deformation curve shows tested protein preparation formed gel is characterized by desirable firmness, and with the highest strength, equal to 152.9 ± 6.29 g. The other main textural parameters of this gel, like consistency, cohesiveness, and viscosity index are given in Table 7 and Figure 4.

The pet food industry employs such substances as proteins, phospholipids, and other surfactants, mostly of natural origin, as emulsifiers. In addition to their gelation ability, the emulsifying properties of any such ingredient are also checked. In general, following the results achieved by Polo et al. [26], it can be stated that more dissociated protein structures and higher net separations between very hydrophilic and lipophilic zones in the polypeptide chain will provide better emulsifying properties. The mussel powder prepared from *S. woodiana* soft tissue was characterized by an emulsifying capacity of more than 230 mL per 1 g of powder. Under the analytical conditions selected for this study. The tested powder showed the tendency to create emulsions, but any comparison to the literature data with respect to this parameter seems unreasonable because of the wide variety of analytical conditions reported by other authors [26,27].

## 4. Conclusions

The work explores the quality properties of the soft tissues of the invasive freshwater mussel *S. woodiana* prepared in the form of freeze-dried powder. The results obtained during this preliminary study, undertaken for the needs of pet food manufacturing, can be concluded thus:

(1) The freeze-dried preparation of soft tissues is a promising source of protein, carbohydrates, ash, and amino acids, including taurine, with an essential role for pets, both cats and dogs,
(2) It is proved that the freeze-drying method reduces the water activity to 0.65 and results in an increase in the content of desirable nutritional ingredients in the final preparation (e.g., protein fraction to circa 35–40% in dry matter). The main proteins determined by electrophoresis (SDS-PAGE) had molecular weights corresponding to actin with a mass of 43–46 kDa.
(3) Furthermore, the *S.* *woodiana* powder had a different bulk density value and a slightly darker color and demonstrated a clear tendency to form relatively stable emulsions.
(4) Despite the relatively low solubility (below 50%) of *S. woodiana* powder determined in this study, gelling properties were observed at 80 °C and a minimum protein concentration of 10.0%, which corresponds well with other protein-based preparations.
(5) Finally, the results obtained show that there is no serious drawback to recommending the preparation examined here as a novel ingredient for pet food type products, although formulas ensuring the best quality of the final product should still be studied; a cost-benefit analysis should be provided before any practical application of powdered soft tissue mussel.

## Figures and Tables

**Table 1:** Microorganism applied in MARA.

Strain No.	Microbial Name	Characteristics
S1	*Microbacterium species*	Gram (+)
S2	*Brevundimonus diminuta*	Gram (−)
S3	*Citrobacter freundii*	Gram (−)
S4	*Comamonastes tosteroni*	Gram (−)
S5	*Enterococcus casseliflavus*	Gram (−)
S6	*Delftia acidovorans*	Gram (+)
S7	*Kurthia gibsonii*	Gram (−)
S8	*Staphylococcus warneri*	Gram (+)
S9	*Pseudomonas aurantiaca*	Gram (+)
S10	*Serratia rubidaea*	Gram (−)
S11	*Pichia anomalia*	Yeast

**Table 2:** Proximate composition and taurine content of fresh and dried soft tissue of *S. woodiana*.

Component (% DM)	Fresh	Freeze-Dried
Moisture	84.89 ± 2.22	10.44 ± 0.11
Ash	15.02 ± 0.99	6.97 ± 0.06
Crude protein	65.07 ± 1.4	34.26 ± 2.22
Lipid	5.69 ± 1.32	2.03 ± 1.01
Carbohydrates	38.97 ± 1.07	52.72 ± 1.96
Taurine ^1^	2064.4 ± 214.69	150.73 ± 44.66

Mean values ± standard deviations (*n* = 3); ^1^ (mg/100 g dry matter).

**Table 3:** Protein and amino acid composition of fish meal and *S. woodiana* mussel powder.

Ingredients	Fish Meal	SW Mussel Powder
Crude protein (% of dry matter DM)	71.40	34.30
Essential amino acids (g per 100 g of protein):		
Alanine	4.28	4.92
Arginine	3.72	7.76
Aspartic acid	6.11	9.48
Glutamic acid	9.18	12.39
Histidine	1.34	2.50
Isoleucine	3.02	4.41
Leucine	5.03	7.73
Lysine	5.03	7.60
Methionine + Cystine	2.25	3.43
Phenylalanine + Tyrosine	4.46	7.64
Proline	2.71	4.90
Serine	2.80	4.30
Threonine	2.77	4.23

**Table 4:** Molecular weight and percentage share of the protein bands marked in *S. woodiana* mussel powder.

Proteins MW		Share of Proteins Band %
No	(kDa)	
1.	>250	13.07 ± 0.20
2.	224–228	4.55 ± 0.02
3.	98–107	11.55 ± 0.51
4.	63	1.73 ± 0.02
5.	43–46	39.51 ± 0.07
6.	38	1.65 ± 0.16
7.	20	9.85 ± 0.14
8.	18	1.94 ± 0.13
9.	16	6.51 ± 0.05
10.	14	1.97 ± 0.30
11.	13	4.91 ± 0.24
12.	10	3.02 ± 0.03

**Table 5:** Physical and physico-chemical characterization of the dried soft tissue of *S. woodiana*.

Parameter	Freeze-Dried *S. woodiana* Preparation
Bulk density (g/mL)	0.313 ± 0.003
Water activity (AW)	0.431 ± 0.005
pH value (10% *w*/*v*)	6.85 ± 0.06
L*	69.493 ± 0.5
a*	23.86 ± 0.10
b*	16.33 ± 0.13
Protein solubility (%)	46.05 ± 0.04
The Least Gelation Concentration (%)	10%
Emulsifying capacity (mL oil/1 g powder)	230 mL ± 15

Mean values ± standard deviations (*n* = 3).

**Table 6:** Least concentration endpoint gelation of *S. woodiana* freeze-dried powder.

Temperature (°C)	Protein Concentration			
	10%	9%	8%	7%
90	+++	++±	−±±	−−−
80	+++	±±+	−−±	−−−
70	++±	±±+	−−±	−−−

+ firm gel forming. ± gel forming tendency. − lack of gel.

**Table 7:** Main textural parameters of gel formed from *S. woodiana* powder diluted in water (10% *w*/*v*).

Parameter	Gels from *S. woodiana* Preparation
Break point (g)	68.4 ± 1.21
Firmness (g)	152.9 ± 6.29
Consistency (g × s)	2868.9 ± 859.27
Cohesiveness (g)	4.2 ± 1.1
Index of viscosity (g × s)	2506.5 ± 365.26

Mean values ± standard deviations (*n* = 3).

## Data Availability

Data sharing not applicable.
